# Basic and clinical immunology – 3014. Pollen lipid can not stimulate iNKT cells in pollen sensitized patients

**DOI:** 10.1186/1939-4551-6-S1-P190

**Published:** 2013-04-23

**Authors:** Mohammad Fereidouni, Shaghayegh Sadat Noorani Hassan Kiadeh, Farahzad Jabbariazad

**Affiliations:** 1 Asthma, Allergy & Immunology Research Center, Birjand University of Medical Sciences, Iran; 2 Immunology, Mashhad Unviersity of Medical Sciences, Mashhad, Iran

## Background

Invariant natural killer T (iNKT) cells are a small subset of T lymphocytes which express a invariant TCR and recognize lipid antigens. They are implicated in a range of immune responses but their role in allergy is controversial. Pollens are the most important trigger of allergic symptoms but their lipid contents may also have a role in allergy through interaction with iNKT cells. The aim of this study was to evaluate the in vitro effect of pollen lipid extract on iNKT cells.

## Methods

PBMCs from 18 pollen-sensitized subjects and 11 healthy controls were isolated and stimulated for 7 days with a mix crude lipid extract of five allergenic pollens prepared by Folch method. Alpha-galactosylceramide and medium were used as positive and negative controls respectively. Proportion of iNKT cells was measured by Flow cytometry using 6B11 and anti-CD3 monoclonal antibodies.

## Results

At day 0, the mean percentage of iNKT cells was 0.42 ± 0.3% and 0.44 ± 0.2% (P > 0.05) for sensitized and control groups respectively. After 7 days culture, iNKT cells from both groups expanded efficiently in response to alpha-galactosylceramide and iNKT proportion reaches to 8 ± 5.6% but lipid extract did not stimulate iNKT proliferation in any groups and the proportion of iNKT cells remained unchanged.

## Conclusions

Based on the results of our study, it seems that pollen lipids are not potent stimulator of iNKT proliferation but further studies need to evaluate the presence and frequency of pollen-specific iNKT cells.

